# The impact of cattle dung pats on earthworm distribution in grazed pastures

**DOI:** 10.1186/s12898-018-0216-6

**Published:** 2018-12-19

**Authors:** M. G. Bacher, O. Fenton, G. Bondi, R. E. Creamer, M. Karmarkar, O. Schmidt

**Affiliations:** 1 Teagasc, Environment Research Centre, Wexford, Ireland; 2 UCD School of Agriculture and Food Science, University College Dublin, Dublin 4, Ireland; 3 Soil Biology Group, Wageningen University, Wageningen, The Netherlands; 4 UCD Earth Institute, University College Dublin, Belfield, Dublin 4, Ireland

**Keywords:** Grassland, Earthworms, Lumbricidae, Soil fauna, Soil biodiversity, Sampling, Spatial distribution, Population aggregation, Populations

## Abstract

**Background:**

Grazed grassland management regimes can have various effects on soil fauna. For example, effects on earthworms can be negative through compaction induced by grazing animals, or positive mediated by increases in sward productivity and cattle dung pats providing a food source. Knowledge gaps exist in relation to the behaviour of different earthworm species i.e. their movement towards and aggregation under dung pats, the legacy effects of pats and the spatial area of recruitment. The present study addressed these knowledge gaps in field experiments, over 2 years, using natural and simulated dung pats on two permanent, intensively grazed pastures in Ireland.

**Results:**

Dung pats strongly affected spatial earthworm distribution, with up to four times more earthworms aggregating beneath pats, than in the control locations away from pats. In these earthworm communities comprising 11 species, temporally different aggregation and dispersal patterns were observed, including absence of individual species from control locations, but no clear successional responses. Epigeic species in general, but also certain species of the anecic and endogeic groups were aggregating under dung. Sampling after complete dung pat disappearance (27 weeks after application) suggested an absence of a dung pat legacy effect on earthworm communities. Based on species distributions, the maximum size of the recruitment area from which earthworms moved to pats was estimated to be 3.8 m^2^ per dung pat. Since actual grazing over 6 weeks would result in the deposition of about 300 dung pats per ha, it is estimated that a surface area of 1140 m^2^ or about 11% of the total grazing area can be influenced by dung pats in a given grazing period.

**Conclusions:**

This study showed that the presence of dung pats in pastures creates temporary hot spots in spatial earthworm species distribution, which changes over time. The findings highlight the importance of considering dung pats, temporally and spatially, when sampling earthworms in grazed pastures. Published comparisons of grazed and cut grasslands probably reached incorrect conclusions by ignoring or deliberately avoiding dung pats. Furthermore, the observed intense aggregation of earthworms beneath dung pats suggests that earthworm functions need to be assessed separately at these hot spots.

**Electronic supplementary material:**

The online version of this article (10.1186/s12898-018-0216-6) contains supplementary material, which is available to authorized users.

## Background

Excluding Antarctica and Greenland, grasslands cover about 40% of the planet’s terrestrial land area [1] and ~ 26% of this area was grazed in 2005 [2]. In many parts of the world pasture based animal agriculture aims to increase both outputs and efficiency through intensification [3, 4]. Such management regimes can have various effects on soil fauna [5–7]. For example, intensification of grazing can have both negative and positive implications for earthworm abundance [8–10]. That is because compaction induced by grazing animals and/or machinery has been shown to limit earthworm activity by interfering with their mobility, feeding and reproduction [11], whereas associated increases in sward productivity and dung pats (or “-patches”) left by grazing cattle provide a food source [12, 13]. Besides domestic grazers on managed pastures, a large variety of wild mammalian grazers and omnivores such as wild boar (*Sus scrofa* L.) spread their dung on natural grasslands [14–16]. Their droppings are equally important for dung fauna, including earthworms, and associated ecological processes such as bioturbation, nutrient cycling and decomposition of organic matter in these natural ecosystems are probably similar to those found in managed ecosystems.

The present work focused on the effects of cattle dung pat deposition on earthworm abundance and distribution within temperate, intensively grazed grassland. Considering the extent of grazed pasture systems, few studies have investigated dung-earthworm interactions and how earthworms aggregate under dung pats. For example, Holter [17] assessed the role of earthworms in dung disappearance in Denmark, while Hendriksen [18] studied dung feeding by detritivorous and geophagous earthworm species. Knight [19] and Svendsen et al. [20] found that earthworm numbers increased up to ten times under dung pats when compared with non-dung pat areas in English pastures. Similarly, James [16] quantified the aggregation of earthworms under bison dung pats in the Tallgrass prairie in Kansas, USA.

Even though all of these studies showed marked differences in earthworm abundance under dung and dung-free areas in grasslands, there is no evidence in the literature that this knowledge has been incorporated into earthworm sampling protocols [8, 13, 21–24] or taken into account in comparative studies of grazed and ungrazed grassland [6, 7]. Spatial earthworm distributions at large scales have been investigated with geo-statistical approaches [12, 25, 26]. At field scale, geostatistics has also been used to describe spatio-temporal earthworm distributions in forest [27] and savannah systems [28]. However, ephemeral resources such as dung pats that are known to affect the distribution of earthworm species have, apparently, not been taken into account. The lack of consideration of dung pats in earthworm sampling schemes is not really a knowledge gap but rather a failure to recognise and implement in protocols a phenomenon that has been documented in a scattered literature. Therefore, the main objective of the present research was to quantify the effect of dung pats on earthworm distributions and to highlight the need for recognition of dung-related patchiness of earthworm distributions when estimating earthworm population sizes and composition in grazed grasslands.

Other knowledge gaps still exist in relation to dung pats and earthworm ecology, including the temporal succession of different earthworm species, reflecting their attraction to dung and their mobility; the legacy effect of dung after decomposition on earthworms; and the effect of dung pats on the spatial dynamic of earthworm populations and their associated functions in soils immediately beneath pats and away from these ‘hot spots’.

The hypotheses of the present study were:

1. Spatial earthworm distribution in grazed grassland is influenced by the aggregation under dung pats of different earthworm species, belonging to different ecological groups.
2. Temporal earthworm arrival at dung pats varies with species, e.g. in a successional manner and re-dispersal as dung pats degrade also varies with species.
3. Soil beneath dung pats keeps supporting larger earthworm populations even after full degradation occurred (legacy effect).

The present study tested these hypotheses using natural and simulated dung pats on two permanent, intensively grazed pasture sites in the SE of Ireland. Results were used to examine and discuss the practical implications in terms of (a) the spatial area of recruitment where earthworms migrate from towards dung pats, and (b) improved earthworm sampling techniques that consider dung pats.

## Results

### Overall earthworm abundance and biomass

**Fig. 1:**
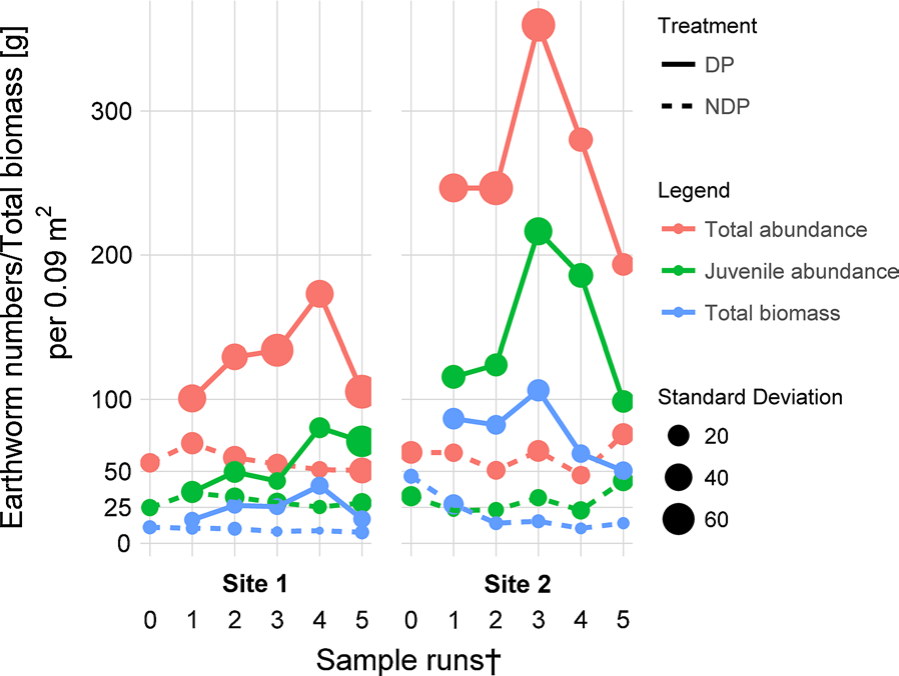
Average earthworm abundance (total and juveniles), as well as total earthworm dead biomass (g) recorded under dung pats (DP) and non-dung pat treatment (NDP). The size of the points indicates the standard deviation per sampling run. ^†^For sampling dates and intervals, please see Table 2

Total-(sum of juvenile and adult earthworms) and juvenile earthworm abundance (Fig. 1) were significantly higher under the dung pat (DP) treatment than under the no-dung pat (NDP) treatment in both experiments (Experiment 1 $\chi^{ 2}_{ 1}$ = 46.9, Experiment 2 $\chi^{ 2}_{ 2}$ = 110.5, p < 0.001 with 1 DF and n = 50). The earthworm abundance peak in DP treatment reached an average of 173 ± 37 individuals per sampling area (0.09 m^2^) in Experiment 1, which translated into a population equivalent of 1900 individuals m^−2^, and 360 ± 60 (Experiment 2) equivalent to 4000 m^−2^. The earthworm biomass (dead weight including gut content) peak reached an average of 40 ± 10 g per sampling area, which translated into a population equivalent of 444 g m^−2^ (Experiment 1) and 106 g ± 20 per sampling area (Experiment 2) equivalent to 1182 g m^−2^.

Considering temporal trends of the overall earthworm abundance, the first sampling after 2 weeks already showed significantly higher abundances under the DP treatment; however, peak abundance, biomass and juvenile abundance under the DP treatment was reached after 7 (Experiment 2; Fig. 1) to 8 weeks (Experiment 1; Fig. 1).

### Experiment specific earthworm abundance

Earthworm abundance under the DP treatment was generally much higher in Experiment 2 than in Experiment 1, while the NDP treatment abundances were similar in the two experiments (Fig. 1). Earthworm abundance under the DP treatment in Experiment 1 ranged (inter quartile range, IQR) from 95 to 164 individuals per sample and in Experiment 2 from 210 to 420 individuals. Control earthworm abundance in both experiments ranged (IQR) from 30 to 80 without significant differences (t test t_24_ = 0.56, p-value = 0.58). Following this trend, earthworm biomass and juvenile abundance under the DP treatment in Experiment 2 were three to five times higher than in Experiment 1, while NDP treatment biomass and juvenile abundance during dung presence were only slightly larger in Experiment 2. From a temporal perspective earthworm biomass and juvenile abundance under dung showed a slower rate of increase in Experiment 1 than in Experiment 2 (Fig. 1).

### Temporal and species effects

Twelve earthworm species were found in total, of which one species was exclusive to each site. The effect of the DP treatment on different species varied in intensity and over time. All ecological groups included species attracted to the DP treatment during one or all sampling runs (except legacy run). Anecic species were generally attracted but low abundances of adults led to less robust results. Endogeic species showed mixed behaviour, with two species showing predominantly attraction and two species showing no clear tendency of attraction or repellence. Epigeic species were mostly attracted to the DP treatment.

**Fig. 2:**
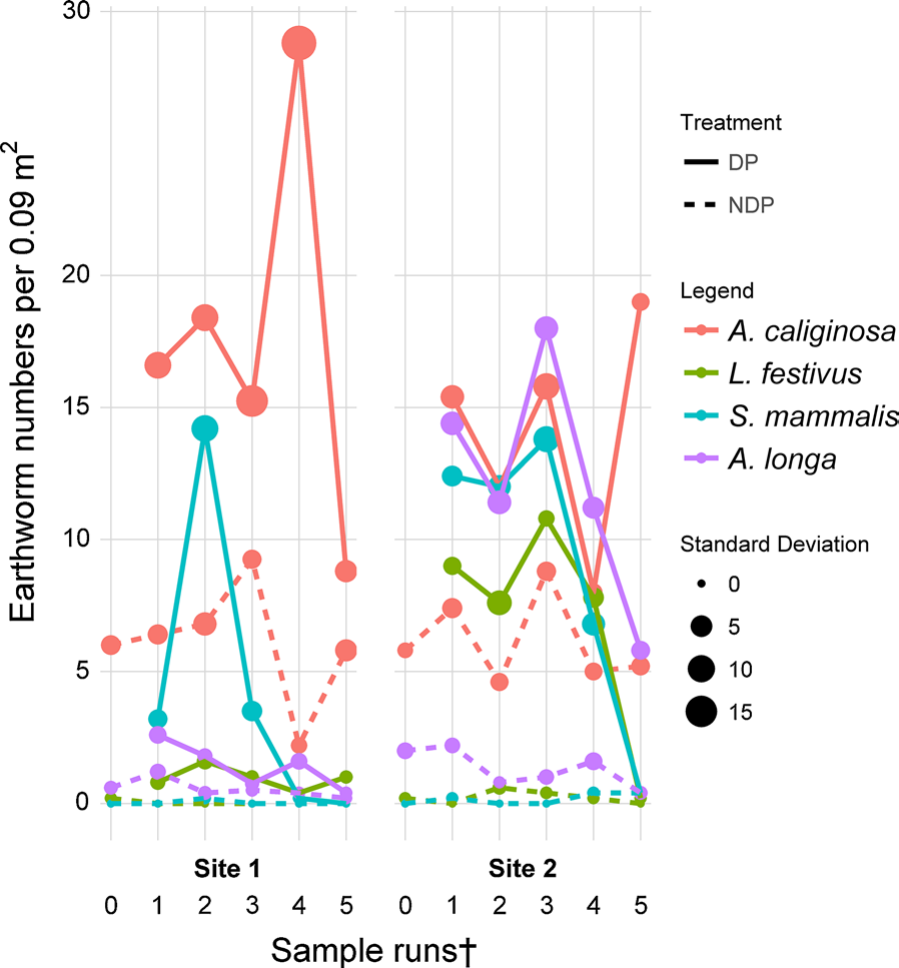
Selected species aggregating under dung pats. Average earthworm abundance of *A. caliginosa* (endogeic)*, L. festivus* (epi-anecic)*, S. mammalis* (epigeic) and *A. longa* (anecic) found per sampling run under dung pats (DP) and non-dung pat treatment (NDP). The size of the points indicates the standard deviation per sampling run. ^†^For sampling dates and intervals, please see Table 2

Two species assigned to the anecic group, *Aporrectodea longa* (Fig. 2) and *Lumbricus friendi,* showed a strong tendency towards the DP treatment in Experiment 2 (*L. friendi*, not shown), while *Lumbricus terrestris* was not clearly attracted. Statistical analysis showed *A. longa* ($\chi^{ 2}_{ 1}$ = 1.853, p > 0.05; $\chi^{ 2}_{ 2}$ = 148.879, p < 0.001) and *L. friendi* ($\chi^{ 2}_{ 1}$ = 0, p = 1; $\chi^{ 2}_{ 2}$ = 77.489, p < 0.001) were significantly attracted to DP treatment in Experiment 2; however, the model fit was low, reported by a very low Akaike information criterion (AIC = 118) compared to other model (AIC = 504 for the general abundance model Experiment 1).

**Fig. 3:**
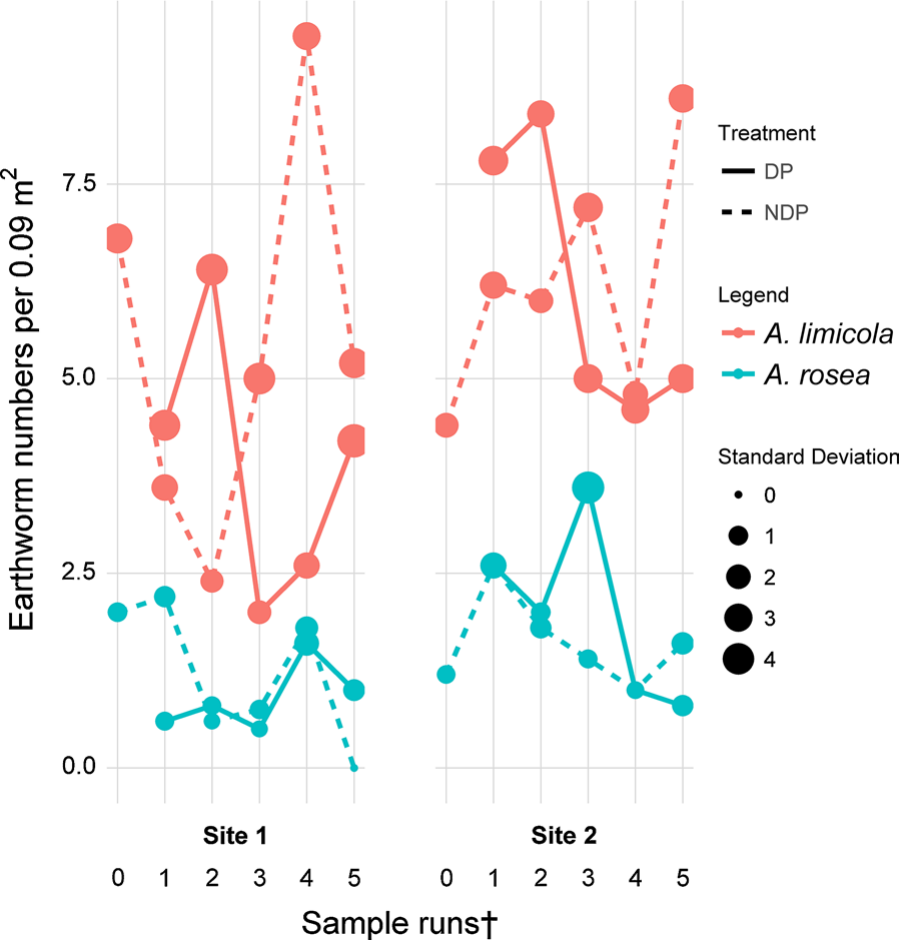
Species not aggregating under dung pats. Average earthworm abundance of *A. limicola* (endogeic) and *A. rosea* (endogeic) found per sampling run under dung pats (DP) and non-dung pat treatment (NDP). The size of the points indicates the standard deviation per sampling run. ^†^For sampling dates and intervals, please see Table 2

The two endogeic species attracted to DP treatment were *Aporrectodea caliginosa* (Fig. 2) ($\chi^{ 2}_{ 1}$ = 4.865, p < 0.05; $\chi^{ 2}_{ 2}$ = 45.446, p < 0.001) and *Allolobophora chlorotica* ($\chi^{ 2}_{ 1}$ = 1.853, p < 0.01; $\chi^{ 2}_{ 2}$ =176.927, p < 0.001). *Aporrectodea limicola* (Fig. 3) and *Aporrectodea rosea* (Fig. 3) showed no general trend of attraction and therefore the models were not able to cover variability of data and were insignificant. *Eiseniella tetraedra* and *Octolasion cyaneum* occurred sporadically only and therefore were not tested statistically.

The epigeic species *S. mammalis* (Fig. 2) was found in large numbers but almost exclusively under the DP treatment ($\chi^{ 2}_{ 1}$ = 24.610, p < 0.001; $\chi^{ 2}_{ 2}$ = 94.087, p<0.001). Similarly, the epi-anecic *L. festivus* (Fig. 2) ($\chi^{ 2}_{ 1}$ = 30.755, p < 0.001; $\chi^{ 2}_{ 2}$ = 136.710, p < 0.001)*, L. castaneus* ($\chi^{ 2}_{ 1}$ = 8.486, p < 0.01; $\chi^{ 2}_{ 2}$ = 49.899, p < 0.001), and *L. rubellus* ($\chi^{ 2}_{ 1}$ = 11.162, p < 0.001; $\chi^{ 2}_{ 2}$ = 35.384, p < 0.001) appeared mainly under DP treatment with statistically significant treatment effects.

The most attracted species moved towards the DP treatment in the first 2 weeks. Of the anecic species, *A. longa* responded quickly, while *L. friendi* responded with higher abundance under DP at the second sampling. The endogeic species *A. chlorotica* and *A. caliginosa* (Fig. 2) were readily attracted at the first sampling and stayed under DP for more than 11 weeks. The epigeic *S. mammalis* and the *L. castaneus* had highest abundances at the second, or third sampling (Fig. 2) and declined thereafter. *S. mammalis* and the epi-anecic *L. festivus* were not recorded after 12 weeks or were only marginally present under DP or NDP treatment.

### Legacy effect

General abundance, biomass and juvenile abundance after 27 weeks were comparable between DP and NDP treatments. The overall abundance and juvenile abundance slightly increased compared to the initial NDP sampling, and the biomass was in the range from previous NDP treatments values. Overall abundance measured in Experiment 2 after 27 weeks (n = 5) was the same in the DP and NDP treatments (no significant differences) (paired test, t_4_ = 0.24, p > 0.05), biomass (paired test, t_4_ = 1.19, p > 0.05) and juvenile abundance (paired test, t_4_ = 0.63, p > 0.05). The endogeic group abundance was the same under the DP and NDP treatments after 27 weeks (mean DP = 25 ± 4, NDP = 22 ± 4); only one species, *A. limicola*, showed a significantly lower abundance under DP (paired t-test, t_4_ = 4.4721, p < 0.05) (abundance mean DP = 4.4 ± 2.0, NDP = 8.4 ± 1.1); *A. longa* was not detected and other species of the anecic group generally showed low abundance. *Lumbricus castaneus* and *L. festivus* of the epigeic and epi-anecic group were not detected either, while *Lumbricus rubellus* and *S. mammalis* (Fig. 2) showed only sporadic abundance.

### Earthworm community

**Fig. 4:**
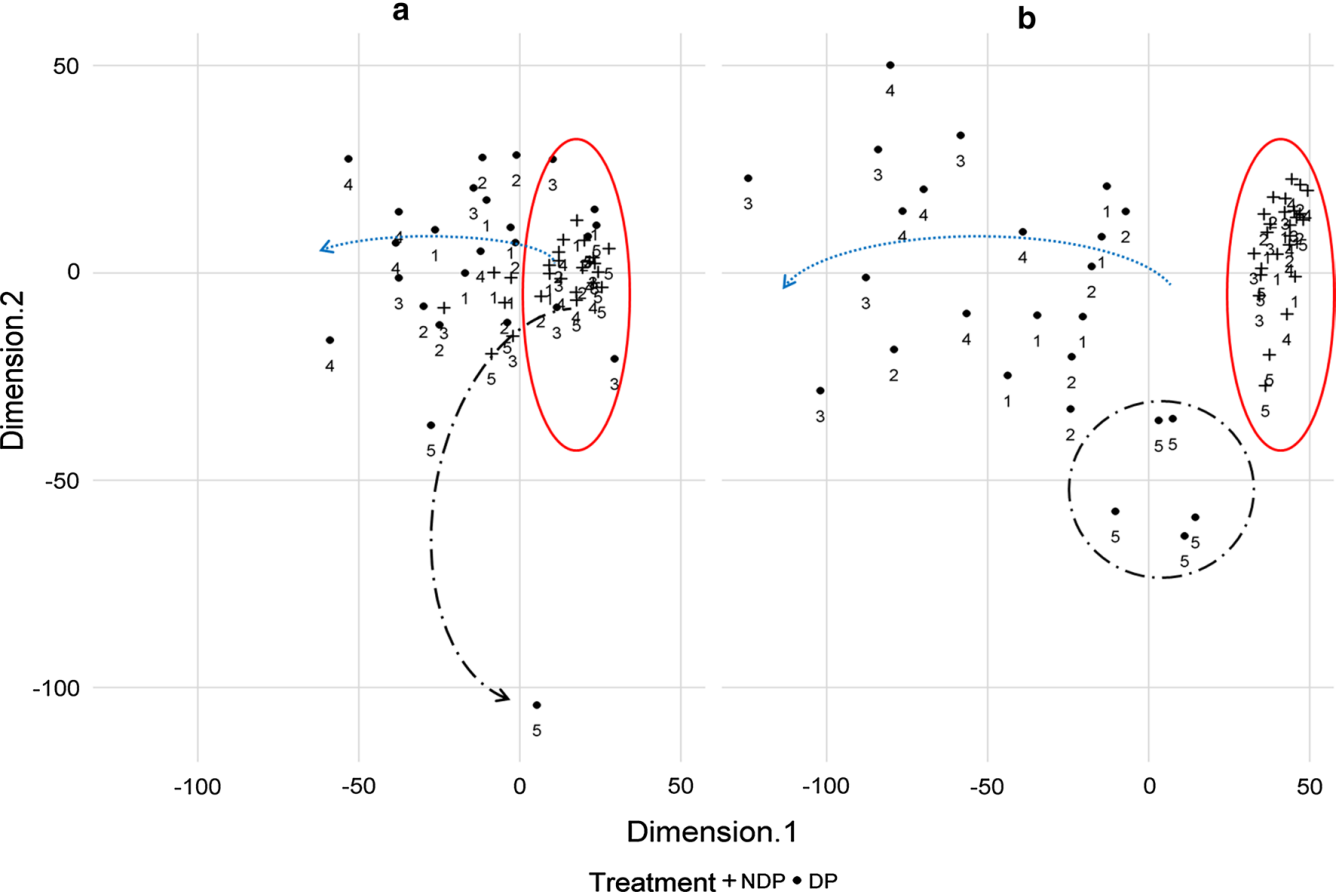
Earthworm communities visualised in a multidimensional scaling plot in **a** Experiment 1 and **b** Experiment 2. Each sampling run is shown as number (see Table 2); Dung pat treatment (DP) observations are shown as dot symbols and no-dung pat treatment (NDP) observations are cross symbols. The trends as indicated by the arrow are pictured in (1) red line ellipse, (2) dotted blue arrow and (3) black long dash dot line (or ellipse)

The development of the community abundance in relation to time was collapsed into a two-dimensional representation for each experiment (Fig. 4a, b). Each point shows multidimensional distances between the species per each observation. For the DP treatment there was a progressive increase of distance in Dimension 1 with the sample run (red arrow). Sampling run 5 (11 weeks) was observed more distant on Dimension 2 than the earlier runs. Treatment NDP showed no marked trends but was very compact. The extent of the trend of the DP treatment in Dimension 1 was more extensive for Experiment 2 (Fig. 4b) than for Experiment 1.

### Recruitment area

The calculated earthworm recruitment radius for dung pats for the total abundance, averaged by sampling run, ranged from 0.2 m to 0.4 m. However, when considering specific species attracted by dung such as the medium-abundant *A. longa* (Fig. 2) the recruitment area increased to up to 0.6 m. For species that had low abundances throughout the pastures such as *L. festivus* that was even absent from most NDP control samples (see Fig. 2), the calculated radius of the recruitment area was up to 1.1 m.

## Discussion

The study’s first hypothesis that spatial earthworm distribution in grasslands is influenced by dung pats was confirmed. The patchy spatial distribution varied also temporally due to differing aggregation and dispersal patterns for individual species, all of which needs to be considered when sampling.

### Earthworm species aggregation

Assessment of earthworm distribution in grazed grasslands needs to specifically take account of aggregation under dung pats. This aggregation effect was shown in both experiments; however, in Experiment 2 considerably higher earthworm abundance and about twice the biomass was recorded under the dung pat treatment than in previous studies in U.K. or Denmark [19, 20]. In terms of species distribution, most epigeic species were aggregating under dung, but also certain species of the anecic and endogeic groups were aggregating under dung pats.

The epigeic species commonly associated with dung [29, 30] showed strong aggregation under dung pats. In particular, *L. castaneus* and *S. mammalis* were nearly exclusively limited to dung locations, reflecting the strong attraction of species of this group to dung pats. *L. festivus* (epi-anecic) (Fig. 2) and the less abundant *L. rubellus* also showed species aggregation.

The endo-anecic designation of *A. longa* (e.g. creation of vertical and horizontal burrows) suggested behavioural flexibility. This explained the results of *A. longa* moving towards and aggregating under dung pats, even though dung was not their first dietary choice in laboratory tests [31]. Aggregation under dung has not been reported before for another species of the anecic group, however in the present study *L. friendi* was attracted to dung pats as well. Aggregation behaviour of the rare species *O. cyaneum* and *E. tetraedra* in this experiment was not conclusive, however the experimental design and effort were aimed at revealing trends for the most abundant species.

Earthworms are attracted to, and therefore aggregate under dung pats. However, certain species could be attracted but are not horizontally mobile e.g. due to permanent burrows of the anecics. Yet, anecic species can travel to dung pats using even soil surface pathways, as mentioned by Knight et al. [19].

Endogeic species *A. chlorotica* and *A. caliginosa* were highly attracted to dung as a food source despite being part of the geophagous group [31]. However, a number of studies [10, 18] assumed that *A. chlorotica* feeds on incorporated and broken down older dung pats, which does not concur with the present observation that *A*. *chlorotica* was abundant under relatively intact dung 2 weeks after deposition. Hendriksen [18] did not record attraction to dung pats by *A. caliginosa* but noted variability in the literature in this regard.

### Spatial effects

The observed aggregation of earthworms under the dung pat treatment means that worms had to travel from the surroundings (here termed ‘recruitment area’) to accumulate under dung pat positions. The estimated recruitment radii of certain species of up to 1.1 m around each dung pat highlights the spatial importance of dung pats at field scale. Mainly for the purpose of greenhouse gas emission research, various studies assessed [32–34] and modelled [35, 36] the extent of grazing cow excreta deposition. It has been estimated that a cow produces 8–12 dung pats a day [36] with an average area of 0.05 m^2^ [33, 34]. Based on these assumptions and the management of Experiment 1, during 6 weeks’ grazing, cows would deposit about 300 dung pats per ha covering directly an area of only 15 m^2^. However, when earthworm abundance was at its peak, the respective recruitment radius considering a single abundant species e.g. *L. festivus* was 1.1 m for each of these dung pats. This, in turn, would translate into a 3.8 m^2^ earthworm influence/recruitment area for each dung pat; however, when multiplied by total pat number, to an area of 1140 m^2^ or about 11% of the total grazing area. Furthermore, since dung pats are deposited repeatedly over time during a grazing season, an even larger influence/recruitment area appears likely until dung pats are completely degraded.

This simple extrapolation emphasizes the likely impact of dung pats on the distribution of earthworms in grazed grasslands. Accounting for this impact by spatial assessment is important in any earthworm study in such systems. Present calculations suggest that certain earthworm species might travel at least 1 m; however, published studies describing patchy earthworm species distribution at larger scales [13, 27, 28] suggests that further travel distances to reach favourable spots such as dung pats are likely.

### Temporal effects

The second hypothesis, regarding temporal arrival and dispersal patterns, was partly confirmed by species moving towards the dung pat in the first 2 weeks; yet, no clear successional response was observed. Knight et al. [19] observed that attraction can occur earlier i.e. within the first 4 days of dung pat deposition. That could be one reason why an obvious successional response to dung pat attraction was generally not observed here because all but *L. friendi* species attracted to dung showed an increased abundance under dung from the first sampling run onwards; competing species with correspondingly decreasing abundance were not identified. Expected successional pattern involving *A. chlorotica* following *L. rubellus* as identified by Murchie et al. [10] was not observed: *A. chlorotica* (also *A. caliginosa*) aggregated already at the first sampling run. However, the experimental design did not allow for examination of this trend before the first sampling. Therefore competition among species for the ephemeral food source as indicated for other dung organisms [37] was not observed. Instead of species succession, *L. castaneus and S. mammalis* showed coexisting behaviour by following similar aggregation and redispersal pattern (in Experiment 1 *L. castaneus* seemed to be one sampling run delayed) from dung pats.

### Legacy effect

The total and juvenile abundance and total biomass observed after 27 weeks did not support the third hypothesis that dung pats have a legacy effect after full degradation. A possible longer-term effect of dung pats on earthworm distributions for over 3 months was discussed by Herrick and Lal [38] for tropical soils. However, the present results based on one sampling date 6 months after dung deposition suggest that anecic and endogeic species did not show a clear preference between previous dung pat locations and no-dung control spots, while epigeic species were largely absent in April, likely due to seasonality [20].

### Earthworm community

The nonmetric multidimensional scaling (nMDS) is used to present ecological effects and communities [39–41]. Up to here earthworm species were discussed separately and over time, whereas nMDS provides an integrated analysis of spatial and temporal trends of the community. The nMDS plot (Fig. 4) illustrates the aggregation of earthworms under dung pats in time, where Dimension 1 can be interpreted as species diversity developing over time, while Dimension 2 somewhat reflected resource exhaustion. In the present study trends were observed, similar to Slade et al. [42] who studied microbial communities associated with dung beetle presence, in relation to dung degradation. Earthworm communities of dung pat and non-dung pat treatments were similar at the beginning, which can be also observed in Slade et al. [42] at the 12 days stage. Communities/treatments diverged then further apart but became eventually more similar. They ended up less distinguishable, agreeing with the more homogenous microbial communities in Slade et al. [42]. Experiment 2 showed a greater spread and distance between treatments and sample runs, which might be caused by more significant differences between treatments by larger earthworm numbers in general. The nMDS plot (Fig. 4) supported the suggestion that controlled conditions in Experiment 2 provided more robust data.

### Implications for earthworm population assessments

To assess earthworm populations, the spatial and temporal aggregation effect of ephemeral resources such as dung pats or droppings has to be considered. Results from the present and previous studies suggest strongly that, in grazed grasslands, earthworm sampling approaches should consider such effects that lead to patchiness of earthworm species on the field scale [5].

Studies on grassland management practices and earthworm abundance that do not consider the above may fall short in terms of reliability of their findings. Studies that compared earthworm populations or distribution of grazed and non-grazed grasslands such as Epelde et al. [6] found a more diverse earthworm community in grassland when grazing was stopped. Similarly, Schlaghamerský et al. [7] compared grazed with mowed grassland sites and found significantly fewer earthworms in grazed grasslands. However, the reported sampling design used could have underestimated earthworm abundances, or even missed specific species such as *S. mammalis* or *L. castaneus.* Comparing the present results with those of Schlaghamerský et al. [7] indicates that most of the species that were less abundant or absent in two of the three pastures of the observed pasture-grassland combinations in Schlaghamerský’s study were highly aggregated below the present dung pat treatment. In another example, Ponge [43] concluded possibly negative effects of higher grazing intensity on soil macro-invertebrate communities without particularly considering the aggregation of earthworms under dung pats. Earlier studies examining the potential of modelling the distribution and abundance of earthworm species in grasslands e.g. *A. caliginosa* did not consider dung pat presence [25]. Reliability of such studies could be improved by combining them with spatial dung pat application models such as that by Yoshitoshi et al. [36].

It is eminently clear from this and previous studies that deliberate sampling away from dung pat areas will likely underestimate some earthworm species or even completely miss specific species such as *S. mammalis* or *L. castaneus* in biodiversity assessments. This is an important point to make for future studies of grazed grassland, grazing intensity gradients or grazing versus non-grazing managements. The following recommendations are offered for such studies:

1. Visually assess dung pat presence and age (consult grazing management records, talk to farmer) on site in question or within a relevant range around the site.
2. Include a minimum or proportional number of dung pat affected locations in the sampling plan. If dung pats are absent at a target site at time of sampling, but present in the vicinity of the site, some dung pats could be sampled qualitatively to determine the absence/presence of ecological groups and species for the site.

## Conclusions

This study addressed knowledge gaps in relation to the behaviour of earthworms towards cattle dung pats in grazed grassland. The study showed that dung pat attraction has temporal and spatial dimensions and that earthworm responses are species specific. The presence of dung pats in pastures creates spatial earthworm species distributions that change with dung pat age. These findings highlight that it is important to consider dung pats, temporally and spatially, when sampling earthworms in grazed pastures and in grasslands with wild mammalian grazers. Published comparisons of grazed and cut grasslands probably came to incorrect conclusions by ignoring or deliberately avoiding dung pats. Furthermore, the observed intense aggregation of earthworms beneath dung pats suggests that earthworm functions need to be assessed separately at these hot spots. Such research is likely to produce more nuanced insights into earthworms as ecosystem engineers that are of wide interest to researchers, farmers and the general public.

## Methods

### Field sites

**Fig. 5:**
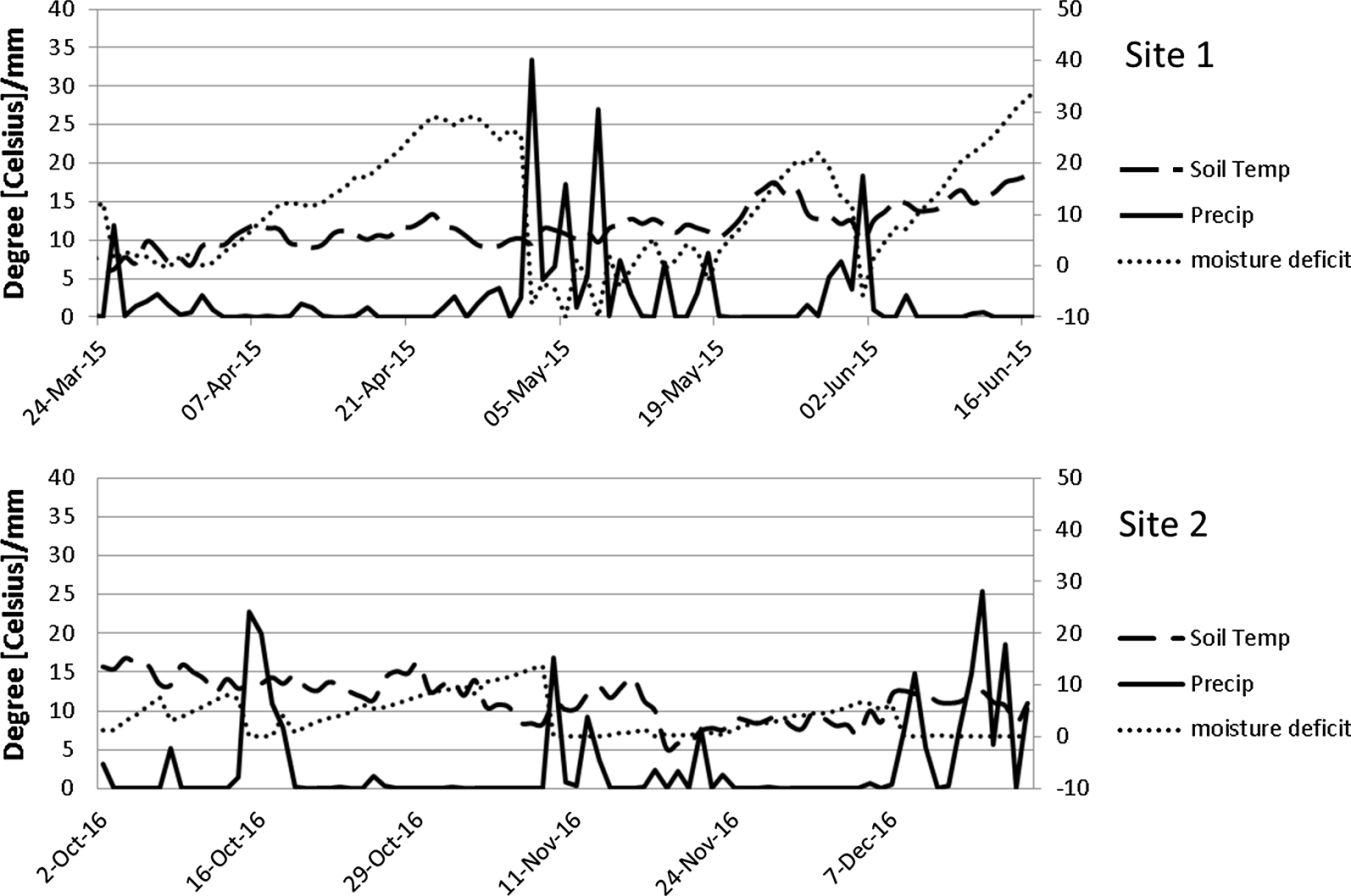
Environmental variables measured on the y-axis (soil temperature, precipitation) or estimated on the alternative y-axis (soil moisture deficit) at the local weather station with dates on the x-axis during Experiment 1 (**a**) and Experiment 2 (**b**)

Two different field-sites (Site 1 and Site 2), located on the dairy research farm of Teagasc, Johnstown Castle, Co. Wexford, Ireland, were selected as representative of intensively managed, permanent grassland. The study area has a mean daily temperature of 9.6 °C and a mean annual precipitation of 1000 mm (effective precipitation is ~ 500 mm) [44]. Air and soil temperature along with precipitation were recorded by the national synoptic weather station situated within the same research centre (within 1 km of both sites) (Fig. 5). Soil moisture deficit (SMD) was estimated using the hybrid grassland model of Schulte et al. [45] based on weather station inputs and assigning a soil drainage class for each site. Two different experiments were carried out: Experiment 1 on site 1 and Experiment 2 on site 2, respectively. Prior to the experiments, intact cores (n = 3) were taken at random to determine bulk density (ρ_b_) [46] and particle size distribution (PSD%) determined by the pipette method [47] of the sites at 5–10 and 10–20 cm depth (core volume 100 cm^3^) for Experiment 1, and at 5–10 cm and 20–25 cm for Experiment 2 (volume 250 cm^3^) (Table 1).

**Table 1:** Soil physical properties (n = 3) for both sites

	Depth (cm)	PSD%			Bulk density
		Sand 2–0.05 mm	Silt 0.05–0.002 mm	Clay < 0.002 mm	Mg m^−3^
Site 1	5–10	68.3	18.7	13.0	1.31 (0.11)
	10–20	n/a	n/a	n/a	1.44 (0.02)
Site 2	5–10	64.4	21.8	13.7	1.22 (0.17)
	20–25	66.2	18.6	15.2	1.46 n/a

*Site 1* Field work at site 1 (0.8 ha, 52.293472, − 6.493222) took place during spring 2015. For this site elevation is 60 m above ordnance datum (m AOD), on a slightly sloped (1%) plain with a south-south-west aspect. The moderately drained soil was classified as stagnic brown earth [48] corresponding to a stagnic cambisol in WRB [49] classification. Soil texture is sandy loam in top and subsoil (20–25 cm). Old red sandstone bedrock is at 15 m depth with average water table position at approximately 2.5 m. The site had been under permanent pasture at least since 2000 with occasional silage cutting and was reseeded in 2008. Intense rotational strip grazing was performed since 2012 with a stocking rate of 1.98 LU ha^−1^ in 2014 (1 Livestock Unit = 1 dairy cow), starting usually in February/March with a 21 day cycle from April to late August/September and then extended to 40 days until October/early November. The site had not been grazed in 2015 before the start of the present experiment. No pesticides or organic fertilizers such as slurry were applied during the experiment, but inorganic fertilizer was applied as shown in Additional file 1. No lime had been applied since 2008. A sward assessment showed predominantly perennial rye grass (*Lolium perenne*).

*Site 2* Field work at site 2 (0.6 ha, 52.29982, − 6.50617) took place during autumn 2016. The site elevation is 80 m AOD, close to the top of a hill and slightly sloped (7%) facing south-south-west. The moderately-drained soil was classified as stagnic brown podzolic [48] which corresponds to a stagnic Podzol within the WRB [49] classification system. The soil texture classification of the top soil and subsoil is sandy loam. Bedrock of Cambrian greywacke is at 10 m depth with an average water table position at 2.5 m depth. The site had not been reseeded since 2007. The management history was regular silage cutting and occasional grazing by dry stock up to 50 days per year with a stocking rate of 1.71 LU ha^−1^ in 2015. The site had not been grazed for the 2016 season and grass was cut before the start of the present experiment. No pesticides or organic fertilizers were applied. Nutrient inputs as inorganic fertilizer are presented in Additional file 1. In 2013, 4.3 t ha^−1^ of calcium lime was applied. A pre-experimental sward assessment showed predominantly perennial ryegrass (*Lolium perenne*), annual meadow grass (*Poa annua*), bent grass (*Agrostis* family), meadow fox-tail (*Alopecurus pratensis*), and mouse-ear chickweed (*Cerastium fontanum*).

### Experimental designs

The experimental designs comprised of naturally deposited cattle dung pats in Experiment 1 and simulated, randomly distributed dung pats in Experiment 2. The two treatments for earthworm sampling were DP: Dung pat treatment, and NDP: No-dung pat treatment (control). Earthworm sampling was conducted (i) at the start of the experiments before dung pat deposition/application; and (ii) over the full life time of dung pats, i.e. from dung deposition/application until disappearance. Sampling was carried out at five dates, once every 2 weeks and 5 spatial replicates were taken on each date for each treatment. To study the influence of soil moisture trends, gravimetric water content was determined at each sampling run following the procedure of Schmidt and Curry [50], whereby a soil sample of 100 g was taken from a depth of 5–15 cm and oven dried at 105 **°**C for 24 h and the weight change recorded.

### Design Experiment 1

The pasture was initially grazed for 4 days in the end of March 2015. Dung pats were naturally deposited by dairy cows at 15.1 LU ha day^−1^ stocking rate (4 days, i.e. each day, one quarter grazed). Thereafter, livestock grazing was avoided during the experiment. Then, 25 dung pats with an approximate diameter of 30 cm were selected and numbered. To avoid field border effects [51], a 10 m margin at the borders of the field was not used. The GPS position of each dung pat was recorded using a Trimble Pathfinder Pro GPS (Trimble Navigation Limited, California, USA) and marked with a magnet to track the location once degradation occurred. For each sampling run a stratified-random pat selection was followed by sampling every fifth DP treatment point. NDP sampling points were selected using a grass area half way between two DP points (see Additional file 2). Distances between dung pats and control plot measurements were at least 10 m. Earthworm sampling was carried out the same way for both experiments (see “Earthworm sampling” section), but due to slow infiltration with no earthworms emerging for two consecutive sampling runs, the allyl isothiocyanate (AITC) treatment was discontinued for Experiment 1.

### Design Experiment 2

Fresh solid dung was collected in September 2016 from dairy cows in the yard and nearby dairy fields over a number of days, avoiding urine and fresh grass residues. Dung was stored at 4 °C for up to 2 weeks and then mixed and homogenised using a drywall mixer. Simulated dung pats (DP treatment) were applied on 3rd of October using a defined fresh weight (2 kg) of dung. Each dung pat had a defined diameter of 30 cm. Dung pats were placed on a plastic mesh (10 mm mesh size) on short mown grass sward, pressed down by their own weight and covered with chicken wire for bird protection. Dung pats were placed in a randomized block design with five blocks (Additional files 2 and 3) each block containing six temporal replicates of the DP treatment and NDP treatment and additional measurements. The distances between sampling blocks were at least 2 m but usually greater (Additional file 3). Sampling intervals were timed as for Experiment 1; however, an additional sampling run was carried out after 6 months (10th April 2017) to test for dung legacy effects (see Table 2).

**Table 2:** Timing of dung pat applications and sampling runs, shown as dates and time elapsed since dung application

Sampling run	Days before or after dung application			
	Days (weeks)	Experiment 1	Days (weeks)	Experiment 2
Initial pre-dung sampling	− 4 (− 1)	24/3/2015	− 1 (0)	02/10/2016
Dung pat application	0 (0)	28/03/2015	0 (0)	03/10/2016
Run 1	14 (2)	10/04/2015	15 (2)	17/10/2016
Run 2	27 (4)	23/04/2015	28 (4)	01/11/2016
Run 3	41 (6)	07/05/2015	51 (7)	22/11/2016
Run 4	55 (8)	21/05/2015	64 (9)	05/12/2016
Run 5	80 (11)	15/06/2015	76 (11)	17/12/2016
Legacy effect run	n/a	n/a	190 (27)	10/04/2017

### Earthworm sampling

To sample under dung pats, the dung pat was visually inspected for earthworms and occurring worms were extracted, then the dung was scratched or lifted off the soil surface. Then, a block of soil of 30 cm × 30 cm × 25 cm depth (0.023 m^3^) was extracted, broken up and manually sorted for earthworms. Hand-sorting was the method of choice to investigate dung effects, however it is known that the relatively small sampling area is less suitable for estimating adult anecic earthworms [50, 51]. An allyl isothiocyanate (AITC) (100 mg L^−1^) solution was poured into the pit to extract earthworms from deeper subsoil layers [52, 53]. Earthworms were collected into a moistened jar, kept in the shade and preserved in formaldehyde solution (4%).

### Earthworm identification

After 8 weeks earthworms were transferred from formaldehyde into 70% industrial methylated spirit for long time storage. Preserved earthworms per sample replicate were blotted dry and weighed (to 0.01 g) on a precision laboratory scale PM200 (Mettler-Toledo LLC, Columbus, OH, USA) to determine the earthworm biomass (with gut content). Mature and sub-adult individuals were identified to species level, juveniles were separated into epilobic un-pigmented, epilobic pigmented and tanylobic earthworms and fragments were recorded separately. An S8APO microscope (Leica Microsystems GmbH, Wetzlar, Germany) and the key by Sims and Gerard [54] were used for identification. Taxonomy follows Sims and Gerard [54].

### Spatial calculations

The potential recruitment area (from where earthworms moved towards a dung pat) was calculated as a recruitment radius using Eq. 1. Total numbers under a dung pat were assumed to be the sum of numbers under grass and the difference having moved in from the surrounding recruitment radius (measured from the centre of the dung pat). In some sampling runs the abundance of certain species under grass was zero, and in such cases the average of that species under grass during the whole experiment was used instead.

1 $$r = \sqrt {\frac{{A_{p} }}{{A_{g} }}\frac{p}{\pi }}$$

where recruitment radius *r*, the dung pat area *p*, the earthworm abundance under the pat A_p_, and the abundance under grass A_g_.

### Statistical analysis

The data were analysed as a factorial combination of treatment (2 levels) and sample run (5 dates). Due to a number of conditions varying between the two experiments (i.e. different site, different year/season, naturally deposited versus simulated dung pats), results were examined separately. The earthworm count data were analysed using a negative binomial model to allow for over-dispersion relative to a Poisson model. The analysis was fitted using the glm.nb function in R [55]. The presence of over-dispersion and residual plots were assessed to ensure that the assumptions of the analysis were met. A type three analysis of deviance (ANOVA) with Chi square test of independence was performed to examine the effect of the treatment. Predicted means were used to compare levels of significant effects. A mixed effect model accounting for the randomized block design applied for Experiment 2 data was used to test for block effects. Sampling runs 1–5 available for both experiments were analysed with the same methods, while the sixth sampling run (Site 2 only) was treated separately. A least square mean comparison applying the Tukey test using the packages lsmeans [56] was used to identify influence of sampling runs on total abundance and species. The development of earthworm communities was assessed using a visualisation based on nonmetric multi-dimensional scaling (nMDS) [57]. The distances between observations consisting of a set of species and each sampling run, in a high dimensional space, was calculated. The nMDS algorithm then plotted the observations in an nMDS plot where distances are represented in an approximate way in 2 dimensions.

## Additional files

**Additional file 1.** Table Fertilization carried out at the study sites during and before Experiment 1 and 2 respectively

**Additional file 2.** Aerial photographs of Experimental Sites 1 and 2 presented at the same scale. Site 1 used for Experiment 1 shows selected dung pat positions (deposited by cows during grazing event) (DP) (solid circles) with control treatment points (NDP) in between (dotted circles) exemplifying only one sampling run (the other 4 sampling runs were omitted for clarity). Site 2 used for Experiment 2 shows dashed lines for each of the 5 replicate blocks along which all dung pats (DP) and treatment control points (NDP) were randomly distributed (Additional file 3). In this case dung pats were simulated by hand.

**Additional file 3.** Dung pat- and sampling layout Experiment 2: Dung pats are indicated with DP and grassland points with NDP. The numbers indicate the sampling runs from 0 “pre-dung pat sampling” to 6 “legacy run”. Sampling points were 0.3 m by 0.3 m, distance between rows was 2 m and horizontal distance was 2.7 m. For each sampling run (1–6), one sample of the DP treatment and one NDP treatment sample were taken from each block (10 in total).
