# Supplementary material for: The impact of cattle dung pats on earthworm distribution in grazed pastures
Source: BMC Ecol. 2018 Dec 19;18:59. doi: 10.1186/s12898-018-0216-6 (PMC6299995; doi:10.1186/s12898-018-0216-6)
Supplement: Supplementary file 1 — Additional file 1. Table Fertilization carried out at the study sites during and before Experiment 1 and 2 respectively [file 12898_2018_216_MOESM1_ESM.pdf]

		Experiment 1	Experiment 2
Date	Fertilizer[a]	kg N ha^{-1}	
3/2/15	25.4	31	
1/4/15	Urea	32	
27/4/15	CAN	24	
14/5/15	CAN	24	
3/6/15	CAN	24	
4/2/16	Urea		32
18/3/16	Urea		40
20/4/16	CAN		32
27/5/16	KAN		30
7/9/16	KAN		30

All figures are in kg of N ha^{-1}

[a]Fertilizers: 25.4 is 25% nitrogen, 4% phosphorus, CAN is calcium ammonium nitrate, KAN is urea with urease inhibitor.
