# Supplementary figures and images for: The impact of cattle dung pats on earthworm distribution in grazed pastures
Source: BMC Ecol. 2018 Dec 19;18:59. doi: 10.1186/s12898-018-0216-6 (PMC6299995; doi:10.1186/s12898-018-0216-6)

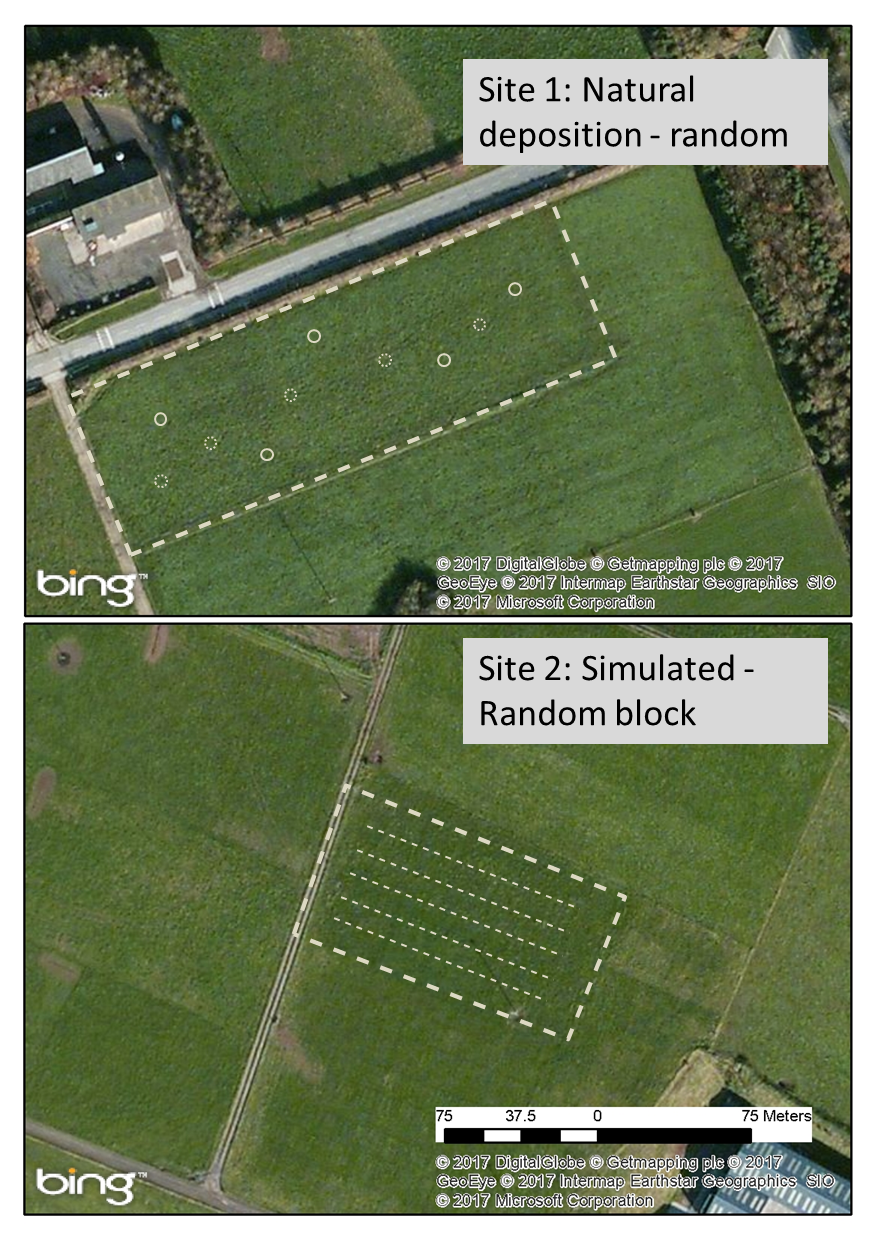

Supplement: Supplementary file 2 — Additional file 2. Aerial photographs of Experimental Sites 1 and 2 presented at the same scale. Site 1 used for Experiment 1 shows selected dung pat positions (deposited by cows during grazing event) (DP) (solid circles) with control treatment points (NDP) in between (dotted circles) exemplifying only one sampling run (the other 4 sampling runs were omitted for clarity). Site 2 used for Experiment 2 shows dashed lines for each of the 5 replicate blocks along which all dung pats (DP) and treatment control points (NDP) were randomly distributed (Additional file 3). In this case dung pats were simulated by hand. [file 12898_2018_216_MOESM2_ESM.png]
