# Supplementary material for: The impact of cattle dung pats on earthworm distribution in grazed pastures
Source: BMC Ecol. 2018 Dec 19;18:59. doi: 10.1186/s12898-018-0216-6 (PMC6299995; doi:10.1186/s12898-018-0216-6)
Supplement: Supplementary file 3 — Additional file 3. Dung pat- and sampling layout Experiment 2: Dung pats are indicated with DP and grassland points with NDP. The numbers indicate the sampling runs from 0 “pre-dung pat sampling” to 6 “legacy run”. Sampling points were 0.3 m by 0.3 m, distance between rows was 2 m and horizontal distance was 2.7 m. For each sampling run (1–6), one sample of the DP treatment and one NDP treatment sample were taken from each block (10 in total). [file 12898_2018_216_MOESM3_ESM.pdf]

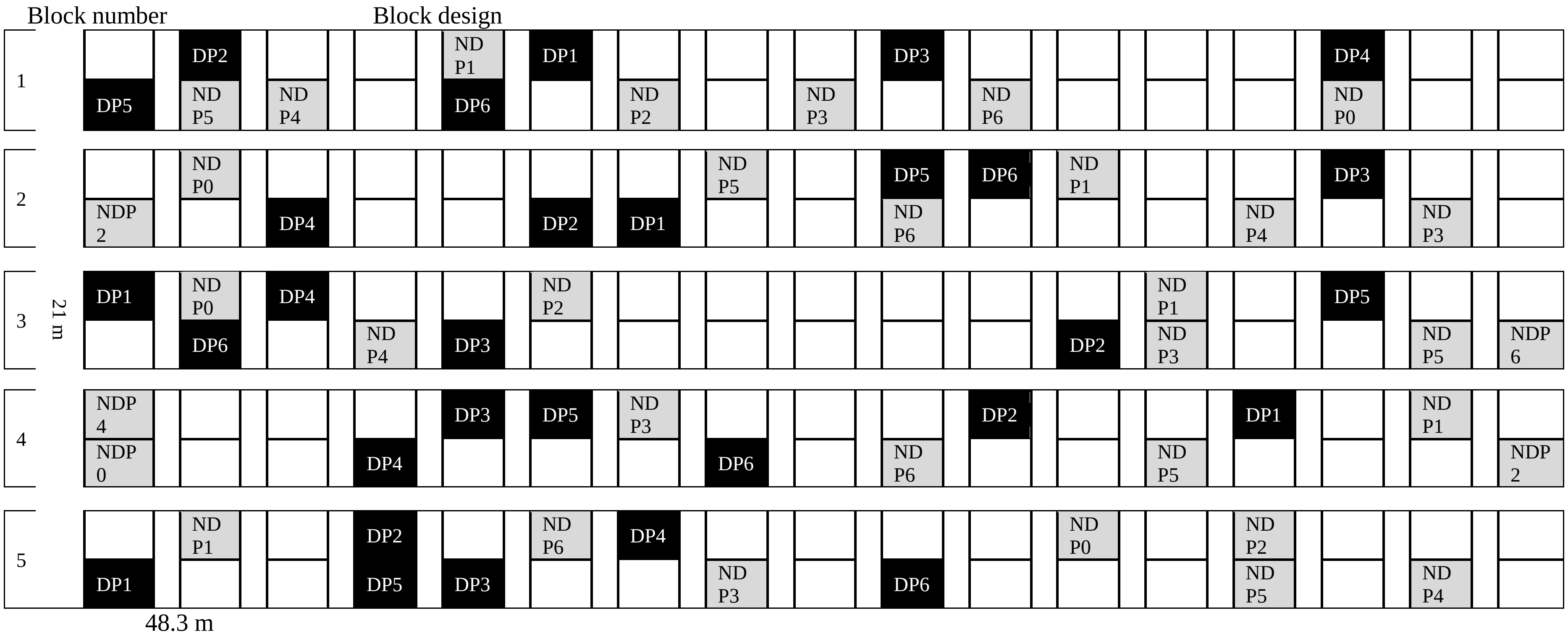
Block number
Block design
1
DP5
DP2
ND P5
ND P4
ND P1
DP6
DP1
ND P2
ND P3
DP3
ND P6
DP4
ND P0
2
NDP 2
ND P0
DP4
DP2
DP1
ND P5
DP5
ND P6
DP6
ND P1
ND P4
DP3
ND P3
3
21 m
DP1
ND P0
DP6
DP4
ND P4
DP3
ND P2
DP2
ND P1
ND P3
DP5
ND P5
NDP 6
4
NDP 4
NDP 0
DP4
DP3
DP5
ND P3
DP6
ND P6
DP2
ND P5
DP1
ND P1
NDP 2
5
DP1
ND P1
DP2
DP5
DP3
ND P6
DP4
ND P3
DP6
ND P0
ND P2
ND P5
ND P4
48.3 m
